# The Influence of an Adrenergic Antagonist Guanethidine (GUA) on the Distribution Pattern and Chemical Coding of Dorsal Root Ganglia (DRG) Neurons Supplying the Porcine Urinary Bladder

**DOI:** 10.3390/ijms222413399

**Published:** 2021-12-13

**Authors:** Paweł Janikiewicz, Barbara Wasilewska, Urszula Mazur, Amelia Franke-Radowiecka, Mariusz Majewski, Agnieszka Bossowska

**Affiliations:** 1 Department of Human Physiology and Pathophysiology, School of Medicine, Collegium Medicum, University of Warmia and Mazury in Olsztyn, Warszawska 30, 10-082 Olsztyn, Poland; pawel.janikiewicz@uwm.edu.pl (P.J.); bachaw@uwm.edu.pl (B.W.); urszula.mazur@uwm.edu.pl (U.M.); mariusz.majewski@uwm.edu.pl (M.M.); 2 Department of Animal Anatomy, Faculty of Veterinary Medicine, University of Warmia and Mazury in Olsztyn, Oczapowskiego 13, 10-719 Olsztyn, Poland; ameliaf@uwm.edu.pl

**Keywords:** guanethidine, urinary bladder, sensory innervation, dorsal root ganglia neurons, immunohistochemistry, neuropeptides, pig

## Abstract

Although guanethidine (GUA) was used in the past as a drug to suppress hyperactivity of the sympathetic nerve fibers, there are no available data concerning the possible action of this substance on the sensory component of the peripheral nervous system supplying the urinary bladder. Thus, the present study was aimed at disclosing the influence of intravesically instilled GUA on the distribution, relative frequency, and chemical coding of dorsal root ganglion neurons associated with the porcine urinary bladder. The investigated sensory neurons were visualized with a retrograde tracing method using Fast Blue (FB), while their chemical profile was disclosed with single-labeling immunohistochemistry using antibodies against substance P (SP), calcitonin gene-related peptide (CGRP), pituitary adenylate cyclase activating polypeptide (PACAP), galanin (GAL), neuronal nitric oxide synthase (nNOS), somatostatin (SOM), and calbindin (CB). After GUA treatment, a slight decrease in the number of FB^+^ neurons containing SP was observed when compared with untreated animals (34.6 ± 6.5% vs. 45.6 ± 1.3%), while the number of retrogradely traced cells immunolabeled for GAL, nNOS, and CB distinctly increased (12.3 ± 1.0% vs. 7.4 ± 0.6%, 11.9 ± 0.6% vs. 5.4 ± 0.5% and 8.6 ± 0.5% vs. 2.7 ± 0.4%, respectively). However, administration of GUA did not change the number of FB^+^ neurons containing CGRP, PACAP, or SOM. The present study provides evidence that GUA significantly modifies the sensory innervation of the porcine urinary bladder wall and thus may be considered a potential tool for studying the plasticity of this subdivision of the bladder innervation.

## 1. Introduction

The storage and periodic elimination of urine requires a complex neural control system that coordinates the activity of a variety of effector organs, including smooth muscles of the urinary bladder [1]. One of the crucial elements of such reflex arc, participating in the regulation of the urinary bladder physiological functions, is dorsal root ganglion (DRG) sensory neurons. Spinal afferent neurons are responsible for transmission of sensations from the bladder, which range from fullness and urgency to discomfort and pain. Working together with sympathetic and parasympathetic efferent nerves, spinal afferent neurons play a key role in reflex control of urinary storage and micturition. Sensory innervation of the urinary bladder has been found to originate in humans, rats, guinea pigs, and cats [2,3,4,5] from the DRG ganglia of the thoraco-lumbar (Th-L; rat, cat, and human), lumbo-sacral (L-S; rat and guinea pig), or sacral (S; cat and human) neuromeres. As reported by Bossowska and collaborators [6], the porcine organ receives dual afferent innervation originating from sensory neurons located in the lumbar (L3-L6) and sacro-coccygeal (S3-S4 and Cq1) DRGs. As the above-mentioned neurons have been shown to use a wide spectrum of transmitters, including substance P (SP), calcitonin gene-related peptide (CGRP), somatostatin (SOM), galanin (GAL), pituitary adenyl cyclase-activating peptide (PACAP), nitric oxide (NO), and calbindin 28 k (CB) [6], it seems likely that differently “coded” sensory neurons, originating from different “centers”, may probably be involved in the regulation of diverse activities of the bladder. The distribution pattern of DRG neurons involved in the urinary bladder innervation is summarized in Table 1.

One of the guanidine derivatives [7], guanethidine (GUA), is known to interfere with adrenergic transmission in the periphery as an antagonist, being used for the first time as an antihypertensive and sympatholytic agent by Maxwell et al. [8]. This selective inhibitor of postganglionic sympathetic transmission [9] acts in rats as a “false” transmitter (causing depletion of norepinephrine-NE from nerve terminals), rather than by inhibiting the association of NE with its receptors on target cells [10]. As of now, two different mechanisms of GUA-mediated inhibition of sympathetic activity have been proposed: first, GUA, transported across the neurolemma by norepinephrine transporter (NET) is accumulating in sympathetic terminals and replacing NE in synaptic vesicles, which leads to a gradual depletion of NE stores in the nerve endings [10] and, in turn, to functional sympathetic denervation [11]. However, Maxwell [12] suggested that GUA first evokes a short-term sympathomimetic effect, caused by a transient release of NE from adrenergic nerve endings, which, in turn, leads to depolarization and stabilization of the neuronal membrane.

Until now, few data have appeared in the available literature concerning the effects of GUA on the sensory nervous system. Supowit and collaborators [13] observed that, in rats, GUA-induced sympathectomy enhanced CGRP and SP mRNA and peptide content in DRG neurons. In contrast, it has been shown that chemical sympathectomy with GUA had no effect on the density of CGRP- and SP-immunoreactive (IR) uterine sensory nerves in rats [14,15]. In urophysiological studies, GUA was mainly used as a drug able to block the effects of sympathetic innervation of the urinary bladder during functional tests examining the influence of various compounds on the contractile activity of this organ in rabbits, cats, guinea pigs, sheep, rats, mice [16,17,18,19,20,21,22], and pigs [23,24,25]. However, there are no relevant data on the influence of GUA on the neuroarchitecture of bladder sensory pathways, while it is well-known that different bladder disorders in humans are associated with several distinct changes in urinary bladder innervation pattern. The neuronal and mechanical events associated with bladder filling and micturition become hypersensitive and progressively painful, and it has been suggested that the detrusor hyperexcitability and pain sensation were mainly due to alterations in the afferent innervation of the organ. Therefore, the purpose of the present study was to investigate, using combined retrograde tract-tracing and immunohistochemistry, the putative influence of intravesical instillation of GUA on the distribution and chemical coding of DRG neurons projecting to the urinary bladder wall. We have decided to use the domestic pig in this experiment since these animals, as opposed to laboratory animals, such as cats and rats, present the advantage that the anatomy, histology, and functionality of their urinary bladder resembles the human one. Therefore, domestic pigs are considered to be an optimal species for preclinical experimentation as they allow for human-related validation of valuable research information gained during the study [26,27].

## 2. Results

### 2.1. Distribution Pattern and Morphometrical Characteristics of Fast Blue-Positive (FB^+^) Neurons in DRG of Control Animals

#### 2.1.1. General Remarks

**Figure 1:**
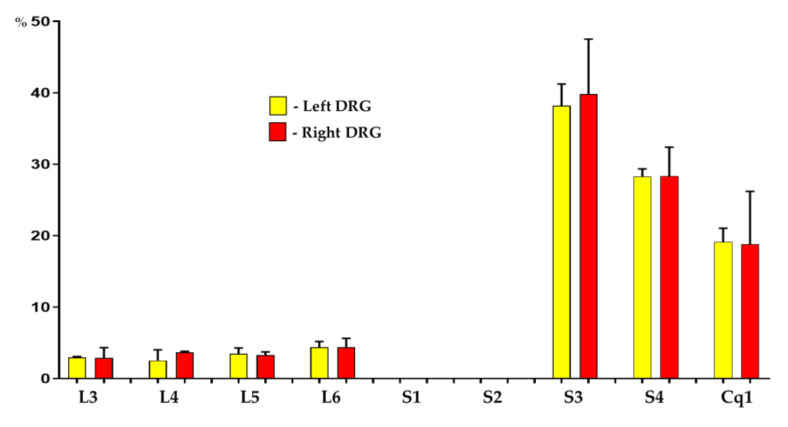
Bar diagram showing the distribution pattern of Fast Blue-positive (FB^+^), bladder projecting cells in the porcine dorsal root ganglia (DRG) ipsi- (red bars) and contralateral (yellow bars) to the sites of tracer injections into the organ wall of control group. Data are pooled and presented as mean ± standard deviation-SD.

Three weeks after the administration of Fast Blue (FB) into the wall of the right side of the urinary bladder, FB-containing sensory neurons were found bilaterally in DRGs of all animals studied. The total number of FB^+^ nerve cells counted in all DRGs per animal ranged from 380 to 540 retrogradely labeled perikarya (454.5 ± 24.8; mean ± standard deviation – SD). Retrogradely labeled cells were found to form two distinct “centers”, located bilaterally in DRGs of the lumbar (L3-L6) as well as sacral (S3-S4) and coccygeal (Cq1) neuromeres of the spinal cord. Urinary bladder-projecting afferent cells were, however, virtually absent from the bilateral S1 and S2 DRGs. The vast majority of traced neurons (85.5 ± 0.9% and 86.9 ± 2.4%; in DRGs on the right and left side of the body, respectively) were located in S3-S4 and in the Cq1 ganglia (S3: 38.2 ± 1.7% and 39.8 ± 4.4%, S4: 28.2 ± 0.6% and 28.3 ± 2.3%, Cq1: 19.1 ± 1.1% and 18.7 ± 4.2%, respectively), while the remainder of FB^+^ nerve cells (14.5 ± 0.9% and 13.07 ± 2.4%) were found in the lumbar ganglia L3-L6 (L3: 2.9 ± 0.1% and 2.8 ± 0.8%, L4: 3.6 ± 0.1% and 2.5 ± 0.8%, L5: 3.4 ± 0.4% and 3.2 ± 0.2%, L6: 4.3 ± 0.4% and 4.4 ± 0.7%, respectively). Details concerning the relative percentages of retrogradely labeled sensory neurons located in the individual right and left DRGs of control group are presented in Figure 1.

It should be stressed that a distinct “lateralization” in the localization of urinary bladder-projecting DRG neurons was observed in terms of their distribution ipsi- and contralaterally to the sites of FB injections into the bladder wall, with a distinct predominance of FB^+^ nerve cells in the ipsilateral ganglia. The number of FB^+^ neurons per animal ranged from 343 to 488 retrogradely labeled perikarya (405.8 ± 22.7) in the DRGs of the right side of the body and from 37 to 58 FB-positive neurons (48.6 ± 2.9) in the left ones. Approximately 89% of all FB-positive spinal sensory neurons (89.3 ± 0.4%) were located in the ipsilateral ganglia, while only 11% (10.7 ± 0.4%) of retrogradely labeled bladder sensory nerve cells was observed in contralateral DRGs.

#### 2.1.2. Morphometrical Characteristics of FB^+^ DRG Neurons Projecting to the Urinary Bladder

Regarding their diameter, FB-positive neurons belonged to all three classes of afferent perikaryal; however, the largest subpopulation of FB^+^ neurons found in the right as well as the left DRGs studied was formed by medium-sized cells with a diameter of 31–50 µm (61.1 ± 1.5% and 58.2 ± 3.2%, respectively). The small-sized bladder sensory neurons (average diameter up to 30 µm) were distinctly less numerous (38.1 ± 1.7% and 40.9 ± 3.3%, respectively), while the “large” sensory neurons (diameter >50 µm) were only occasionally found in the DRG studied (0.8 ± 0.2% and 0.9 ± 0.1%, respectively).

Differences in the numbers of individual “size classes” of sensory cells were observed between the DRGs of L and S-Cq neuromeres: while in the ganglia of lumbar region most of the cells belonged to the population of “medium” (61.7 ± 1.5%) and “small” (38.3 ± 1.5%) neurons (“large” cells were not observed at all), in the DRG of S and Cq segments, labeled cells mainly belonged to the population of “small” neurons (59.0 ± 4.0%); “medium”-sized neurons (39.5 ± 4.2%) and “large” cells (1.5 ± 0.2%) constituted smaller populations, respectively. 

#### 2.1.3. Intraganglionic Distribution Patterns of Traced Cells

**Figure 2:**
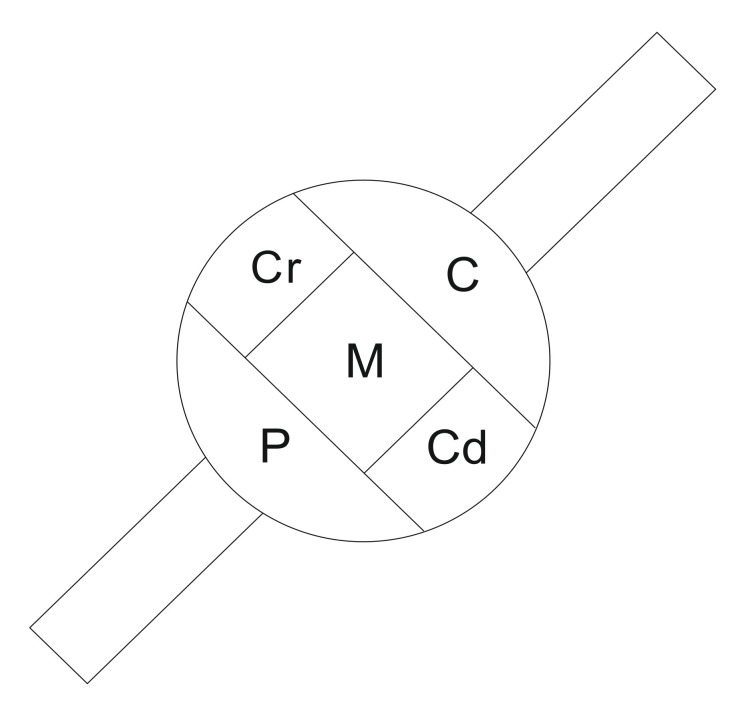
A schematic drawing of a spinal ganglion section showing its arbitrary division into topographical subdomains, in which the occurrence and relative frequency of FB-containing urinary bladder-projecting neurons (UB-PNs) were studied. C—central, P—peripheral, Cr—cranial, and Cd—caudal domains of the DRG, M—middle region of the ganglion.

To establish the pattern of FB^+^ cell distribution in the DRGs tested, the “mask” shown in Figure 2 was applied to each ganglionic section analyzed. UB-PNs were present in all five subdomains of sensory ganglia studied, although a distinct accumulation of the retrogradely labeled cells was observed in the caudal (35.9% ± 1.4%) and cranial parts (30.8% ± 1.2%) of the DRG studied, while lower numbers of FB^+^ neurons were observed in the central, middle, and peripheral regions of the ganglia (Figure 2; 12.3% ± 0.8%, 9.4% ± 0.7% and 11.6% ± 2.3%, respectively). 

The distribution pattern of FB^+^ cells in the DRGs contralateral to the site of tracer injections was very similar to that seen in the ipsilateral ones: the majority of FB^+^ nerve cell bodies were present in the caudal and cranial subdomains (37.03 ± 2.5% and 33.1 ± 2.4%, respectively), with the rest of FB^+^ perikarya dispersed quite evenly in the central, middle, and peripheral regions of the ganglion (10.5 ± 0.6%, 10.8 ± 1.3%, and 8.5 ± 1.4%, respectively).

### 2.2. Distribution Pattern and Morphometrical Characteristics of FB^+^ Neurons in the DRG of GUA-Treated Pigs

#### 2.2.1. General Remarks

The distribution patterns as well as the morphometrical characteristics of FB^+^ neurons in animals not only injected with retrograde tracer but also exposed to GUA were very similar to those described above. Briefly, the total number of FB^+^ nerve cells per animal ranged from 373 to 536 retrogradely labeled perikarya (447.3 ± 25.9), and they were bilaterally distributed, with a clear ipsilateral prevalence. In the right ganglia, the number of FB-containing neurons per animal ranged from 335 to 487 retrogradely labeled perikarya (397.5 ± 35.7) and in the left ganglia from 38 to 49 FB-positive neurons (44.0 ± 2.3). Approximately 90% of all FB-positive spinal sensory neurons (89.8 ± 0.5%) were located in the ipsilateral ganglia. Only about 10% (10.2 ± 0.5%) of retrogradely labeled bladder sensory nerve cells were observed in contralateral DRGs.

**Figure 3:**
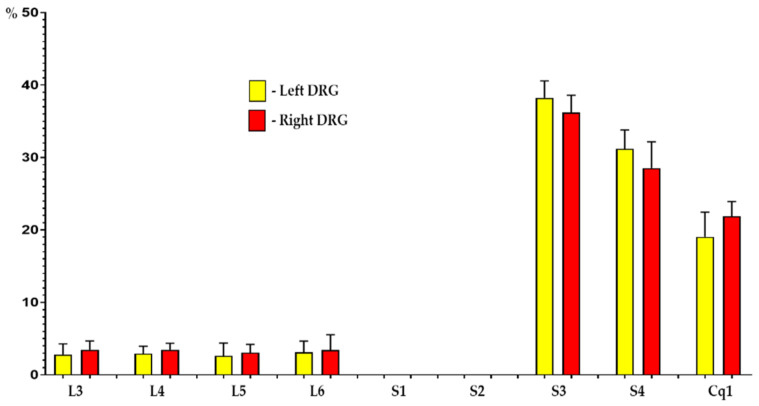
Bar diagram showing the distribution pattern of FB-positive, bladder projecting cells in the porcine DRG ipsi- (red bars) and contralateral (yellow bars) to the sites of tracer injections into the organ wall of guanethidine (GUA)-treated animals. Data are pooled and presented as mean ± SD.

As in the control group, retrogradely labeled sensory neurons supplying the urinary bladder formed two separate “nerve centers” located in the DRGs of the L3-L6 as well as of the S3-Cq1 segments of the spinal cord. Furthermore, in both the ipsilateral and the contralateral ganglia, the vast majority (86.6 ± 3.0% and 88.5 ± 3.2%) of sensory neurons supplying the porcine urinary bladder were located in the sacral and coccygeal ganglia (S3: 36.2 ± 1.4% and 38.2 ± 1.3%, S4: 28.5 ± 2.1% and 31.2 ± 1.5%, Cq1: 21.9 ± 1.2% and 19.1 ± 1.9%, respectively). The remainder of FB^+^ nerve cells (13.4 ± 3.0% and 11.5 ± 3.2%) were found in the lumbar ganglia from L3 to L6 (Figure 3; 3.4 ± 0.7% and 2.8 ± 0.8%, 3.4 ± 0.5% and 2.9 ± 0.5%, 3.1 ± 0.6% and 2.6 ± 1.0%, 3.5 ± 1.2% and 3.2 ± 0.8%, respectively). Details concerning the relative percentages of retrogradely labeled sensory neurons located in the individual right and left DRGs of GUA-treated animals are presented in Figure 3.

#### 2.2.2. Morphometrical Characteristics of FB^+^ DRG Neurons Projecting to the Urinary Bladder

Regarding cell diameters, the most numerous subpopulation of FB^+^ neurons found in the right as well as in the left DRGs included medium-sized cells (59.4 ± 1.5% and 59.2 ± 2.9%). Small-sized neurons were less numerous (39.5 ± 1.5% and 39.9 ± 2.8%), while the “large” FB-containing perikarya were only occasionally observed (1.1 ± 0.1% and 0.9 ± 0.1%). In the lumbar DRGs, most FB^+^ neurons were medium- (60.7 ± 2.1%) and small-sized (39.3 ± 2.1%) cells; however, large sensory neurons were not found. In sacral and coccygeal DRGs, FB^+^ cells were mainly of small diameter (59.2 ± 1.6%), while medium-sized neurons were less numerous (39.8 ± 1.7%). “Large” cells (1.0 ± 0.1%) were only sporadically seen.

#### 2.2.3. Intraganglionic Distribution Patterns of Traced Cells

The intraganglionic distribution pattern of urinary bladder-projecting neurons (UB-PNs) within both the ipsilateral and contralateral DRGs in the GUA-treated animals was similar to this observed in the control group. In both the ipsi- and contralateral ganglia, a distinct accumulation of retrogradely labeled cells was found in the caudal (38.3% ± 1.2% and 41.5 ± 1.0%) and in the cranial subdomain (36.3% ± 1.2% and 34.6 ± 1.3%), while a lower number of FB^+^ sensory neurons was observed in the central, middle, and peripheral regions of studied ganglia (10.2% ± 0.8% and 8.9 ± 0.8%, 9.1% ± 0.6% and 7.5 ± 0.7%, 6.1% ± 1.2% and 7.5 ± 2.4%, respectively). No significant differences in the number, diameter, or distribution pattern of FB-positive neurons in both the right and the left DRG ganglia were observed between the control and the GUA-treated group.

### 2.3. Immunohistochemical Characteristics of FB^+^ Neurons in the DRG of Control and GUA-Treated Animals

Both in the group of control animals as well as in those treated with GUA, the use of the single-immunofluorescence technique demonstrated that, in the studied DRGs, about 2/3 of the FB^+^ neurons contained any of the tested biological active substances (SP, CGRP, PACAP, GAL, neuronal nitric oxide synthase—nNOS, SOM, or CB; 64.2 ± 1.6% vs. 67.1 ± 3.3%, control vs. GUA-treated group, respectively), while about 1/3 were immunonegative to all antisera used (35.8 ± 1.6% vs. 32.9 ± 3.3%, respectively).

After GUA instillation, there were no statistically significant differences in the number of UB-PNs containing particular neurotransmitters (67.1 ± 3.3%) as well as in the number of FB^+^ nerve cells without any biological active substances studied (32.9 ± 3.3%) compared to the group of healthy animals. 

#### 2.3.1. SP-containing UB-PNs in the DRG of Control and GUA-Treated Animals

**Figure 4:**
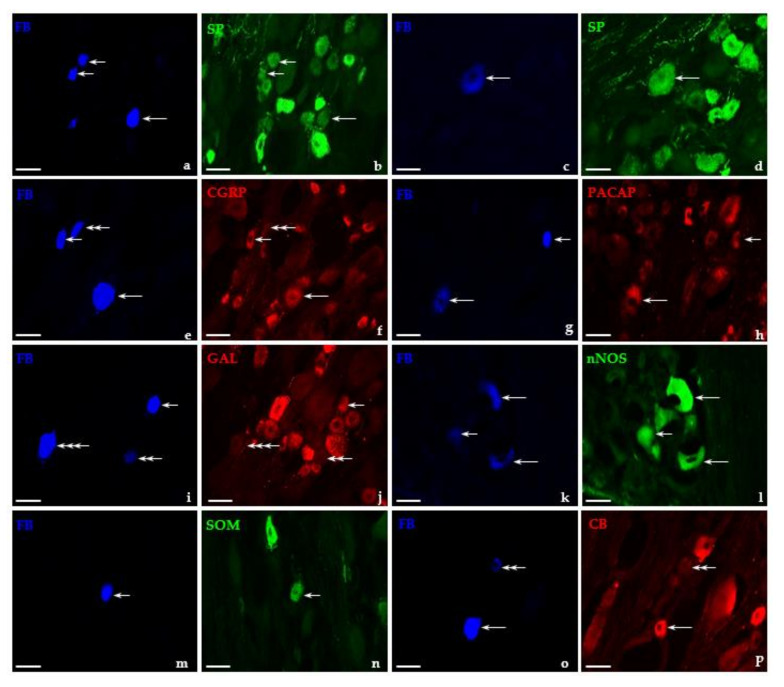
Representative images of DRG-UB-PNs in the control pigs. All the images were taken separately from blue (**a,c,e,g,i,k,m,o**), green (**b,d,l,n**), and red (**f,h,j,p**) fluorescent channels. Short arrows represent small-sized FB-positive DRG-UB-PNs (**a,e,g,i,k,m**) which were simultaneously substance P- (SP; **b**), calcitonin gene-related peptide- (CGRP; **f**), pituitary adenylate cyclase-activating polypeptide- (PACAP; **h**), galanin- (GAL; **j**), neuronal nitric oxide synthase- (nNOS; **l**) or somatostatin-positive (SOM; **n**). Long arrows represent middle-sized FB-positive DRG-UB-PNs (**a,c,e,g,k,o**) that simultaneously contained SP (**b**), CGRP (**f**), PACAP (**h**), nNOS (**l**), or calbindin (CB; **p**). Double arrows represent small-sized FB-positive DRG-UB-PNs (**e,i,o**), which were simultaneously CGRP- (**f**), GAL- (**j**), or CB-negative (**p**). Triple arrows represent middle-sized FB-positive DRG-UB-PN (**i**), which was simultaneously GAL-negative (**f**). Bar in all the images—50 μm.

In general, under physiological conditions, the largest subpopulation of FB-positive DRG neurons was that containing SP (Figure 4a,b; 45.6 ± 1.3%). SP^+^ sensory nerve cells were present both in the lumbar (44.6 ± 2.1%) as well as in the sacral/coccygeal (45.4 ± 1.3%) population of retrogradely labeled cells, and there were no significant differences in the number of UB-PNs immunolabeled for SP in these two bladder-projecting “centers”.

**Figure 5:**
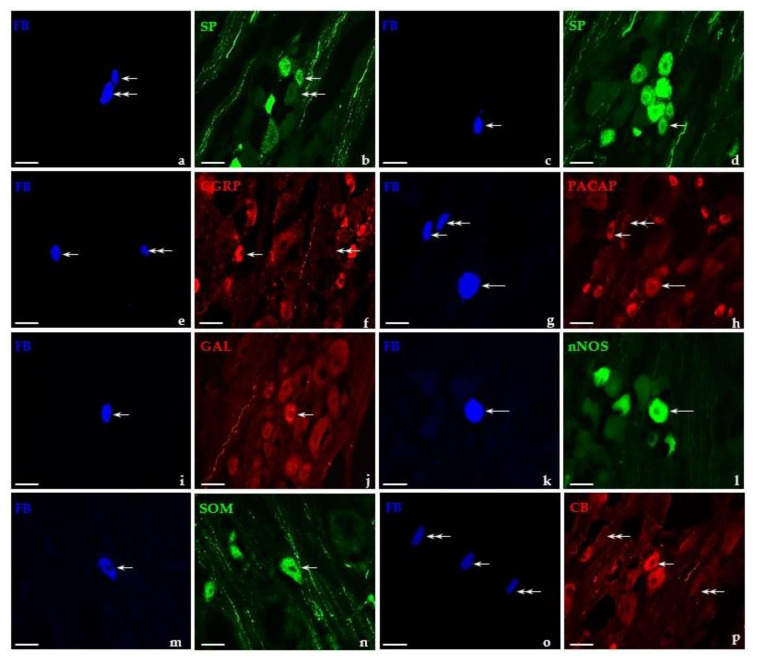
Representative images of DRG-UB-PNs in the GUA-treated pigs. All the images were taken separately from blue (**a,c,e,g,i,k,m,o**), green (**b,d,l,n**), and red (**f,h,j,p**) fluorescent channels. Short arrows represent small-sized FB-positive DRG-UB-PNs (**a,c,e,g,i,m,o**) which were simultaneously SP- (**b,d**), CGRP- (**f**), PACAP- (**h**), GAL- (**j**), SOM- (**n**), or CB-positive (**p**). Long arrows represent middle-sized FB-positive DRG-UB-PNs (**g,k**), which simultaneously contained PACAP (**h**) or nNOS (**l**). Double arrows represent small-sized FB-positive DRG-UB-PNs (**a,e,g,o**), which were simultaneously SP- (**b**), CGRP- (**f**), PACAP- (**h**), or CB-negative (**p**). Bar in all images—50 μm.

After GUA instillation, a slight decrease in the “total” number of FB^+^/SP^+^ neurons (Figure 5a–d; 34.6 ± 6.5%) was observed; however, it should be stressed that, in comparison with the control group, such diminution was only observed in the sacral/coccygeal subpopulation (10.8 ± 2.9%), while in the lumbar DRGs, the number of FB^+^/SP^+^ cells did not significantly change (42.6 ± 2.8%).

Regarding their diameter, FB/SP-positive neurons of untreated animals belonged to two classes of afferent perikarya: the most numerous subpopulation of small-sized cells (Figure 4a,b-short arrows; 83.4 ± 0.4%) and distinctly less numerous subset of medium-sized perikarya (Figure 4a–d-long arrows; 16.6 ± 0.4%). In GUA-treated animals, a significant decrease in the number of small-sized SP^+^ bladder-projecting neurons (Figure 5a–d-short arrows; 60.9 ± 4.4%) was observed, while simultaneously the number of medium-sized FB^+^/SP^+^ neurons (39.1 ± 4.4%) distinctly increased. SP^+^ “large” retrogradely traced neurons were not found either in the control or in GUA-challenged animals.

In both the control and GUA-treated animals, the majority of FB^+^/SP^+^ neurons were unevenly distributed, as isolated cells scattered throughout the individual ganglionic subdomains. Only a few SP^+^ UB-PNs of the untreated animals were found to form small, loose clusters of up to three neurons. In both groups, a distinct accumulation of the retrogradely labeled SP-positive cells was found in the caudal (32.2 ± 2.2% vs. 32.6 ± 1.5%) and in the cranial ganglionic region (28.5 ± 1.8% vs. 29.7 ± 1.2%), while such coded cells were less numerously present in the central, middle, and peripheral part of the ganglia (14.2 ± 1.6% vs. 15.1 ± 0.7%, 12.6 ± 1.0% vs. 12.9% ± 0.7% and 12.5 ± 0,9% vs. 9.6 ± 1.0%, respectively).

#### 2.3.2. CGRP-Containing UB-PNs in the DRG of Control and GUA-Treated Animals

CGRP was found in 35.6 ± 1.6% (Figure 4e,f) of all retrograde traced DRG neurons in the untreated group, and there were no distinct changes in the number of CGRP-IR FB^+^ neurons (Figure 5e,f; 38.6 ± 0.8%) after GUA treatment. Furthermore, there were significantly more FB^+^/CGRP^+^ neurons in the lumbar DRGs (48.5 ± 1.4% vs. 52.3 ± 0.5%) compared to the sacral/coccygeal ganglia studied (29.2 ± 2.0% vs. 28.5 ± 1.2%) in the untreated vs. GUA-treated animals, respectively.

In general, CGRP-IR was observed in all three “size classes” of afferent perikarya of control animals, being present in 60.4 ± 1.6% of “medium” (Figure 4e,f-long arrows), 31.7 ± 1.6% of “small” (Figure 4e,f-short arrows), and 7.9 ± 0.9% of “large” cells, respectively. GUA instillation led to a significant increase in the number of small-sized FB^+^/CGRP^+^ neurons (Figure 5e,f-short arrows; 74.2 ± 2.8%), while the number of medium-sized subpopulation of CGRP^+^ perikarya (25.8 ± 2.8%) distinctly decreased; in addition, the complete disappearance of “large” FB^+^/CGRP^+^ cells was observed. 

Although, in both the control and GUA-treated animals, FB^+^/CGRP^+^ neurons were unevenly scattered throughout the individual ganglionic sections, a distinct accumulation of these cells was found in the cranial (46.5 ± 0.9% vs. 47.8 ± 1.2%) and the caudal ganglionic domains (44.3 ± 1.3% vs. 45.6 ± 2.1%); on the other hand, only a few CGRP^+^ bladder-projecting cells were observed in the central, middle, and peripheral regions of the ganglia (4.4 ± 0.4% vs. 3.2 ± 0.9%, 2.8 ± 0.2% vs. 2.1% ± 0.7% and 2.0 ± 0,9% vs. 1.3 ± 1.0%), respectively.

#### 2.3.3. PACAP-Containing UB-PNs in the DRG of Control and GUA-Treated Animals

Under physiological conditions, the third largest population of FB-positive neurons in all DRGs studied were those containing PACAP (Figure 4g,h; 26.7 ± 1.6%), and this value did not change significantly after GUA-instillation (Figure 5g,h; 29.9 ± 1.0%). Furthermore, the sacral/coccygeal DRGs contained a significantly higher number of PACAP-positive retrogradely traced nerve cells (32.9 ± 0.9% vs. 30.4 ± 1.4%) in both groups of animals when compared to the lumbar ganglia (23.2 ± 1.0% vs. 25.9 ± 0.3%).

In untreated animals, PACAP-positive UB-PNs belonged mainly to the subpopulation of small-sized cells (Figure 4g,h-short arrows; 80.9 ± 4.8%), while the number of medium-sized neurons (Figure 4g,h-long arrows; 19.1 ± 4.8%) was distinctly smaller. Differences in the numbers of both small PACAP-IR bladder-projecting sensory neurons (Figure 5g,h-short arrows; 81.4 ± 2.7%) and those of the medium size (Figure 5g,h-long arrows; 18.6 ± 2.7%) were statistically insignificant after intravesical administration of GUA.

The intraganglionic distribution pattern of FB^+^/PACAP^+^ neurons in DRGs studied was very similar in both the control and GUA-treated animals: the largest number was observed in the caudal region (53.7 ± 2.9% vs. 52.3 ± 3.2%), then in the cranial domain (30.2 ± 0.8% vs. 33.2 ± 2.1%), while in the middle, peripheral, and central domains of DRGs, only single PACAP-positive nerve cells were present (7.9 ± 0.9% vs. 6.9 ± 1.9%, 4.8 ± 1.5% vs. 4.6 ± 2.4% and 3.4 ± 1.2% vs. 3.0 ± 1.1%, respectively).

#### 2.3.4. GAL-Containing UB-PNs in the DRG of Control and GUA-Treated Animals

In untreated animals, a small population of FB^+^ DRG neurons expressed GAL-IR (Figure 4i,j; 7.4 ± 0.6%), while the intravesical administration of GUA led to a statistically significant increase in the number of GAL-containing UB-PNs (Figure 5i,j; 12.3 ± 1.0%). While in the control animals, the number of GAL-IR FB^+^ neurons was distinctly higher in the lumbar than in the sacro-coccygeal DRGs (12.2 ± 1.0% vs. 5.6 ± 0.5%), instillation of the drug led to a reversal of the situation: 8.7 ± 0.3% of FB^+^ cells in the lumbar and 15.9 ± 0.9% in the sacral/coccygeal DRGs expressed GAL-IR after this treatment. 

In both the control and GUA-treated group, FB/GAL-positive nerve cells belonged exclusively to the small-sized type of sensory neurons (Figure 4i,j and Figure 5i,j -short arrows, respectively). In general, the distribution of FB^+^/GAL^+^ neurons were virtually restricted to only two domains of DRG studied in control animals: the cranial and the caudal regions (59.6 ± 2.0% vs. 40.4 ± 2.0%, respectively). This pattern of intraganglionic distribution was basically repeated in animals treated with GUA (59.4 ± 1.5% vs. 38.4 ± 1.6%, cranial vs. caudal domains), but it should be emphasized that, in this group, single FB^+^/GAL^+^ cells appeared also in both the middle (1.3 ± 0.1%) and the central (0.9 ± 0.1%) ganglionic domains.

#### 2.3.5. nNOS-Containing UB-PNs in the DRG of Control and GUA-Treated Animals

nNOS was found in 5.4 ± 0.5% of all retrogradely traced neurons in DRGs of animals of control group (Figure 4k,l). The lumbar DRGs contained a significantly higher number of nNOS-positive retrogradely traced cells (10.7 ± 0.9%) than sacral/coccygeal ganglia (2.3 ± 0.3%). A distinct increase in the total number of FB^+^ neurons immunolabeled for nNOS (Figure 5k,l; 11.9 ± 0.6%) was found after GUA treatment; however, it should be stressed that this increase (to 9.0 ± 1.3%) was primarily observed in the sacro-coccygeal DRGs, while in the lumbar DRGs, the vGUA-treatment-evoked increase in the number of nNOS-positive retrogradely labeled perikarya was less pronounced (14.9 ± 0.2%).

Regarding their diameter, FB/nNOS-positive neurons of control animals belonged to two classes of afferent perikarya. The most numerous subpopulation of FB^+^/nNOS^+^ neurons found in all DRGs studied was the medium-sized cells (Figure 4k,l-long arrows; 65.1 ± 2.0%), while the small-sized nitrergic cells were found to be distinctly less numerous (Figure 4k,l-short arrows; 34.9 ± 2.0%). GUA instillation led to a significant increase in the number of small-sized nNOS^+^ bladder-projecting neurons (73.7 ± 3.6%), with a concomitant decrease in the number of medium-sized FB/nNOS-positive cells (Figure 5k,l-long arrows; 26.3 ± 3.6%).

In both experimental groups, nNOS^+^ neurons were arranged individually inside the ganglion, mainly in its caudal (58.5 ± 3.3% vs. 55.5 ± 3.8%) and cranial (36.6 ± 3.8% vs. 39.3 ± 3.4%) regions. The remainder of nNOS-containing, retrogradely labeled nerve cells were present in the central (3.6 ± 0.4% vs. 4.0 ± 0.8%) and middle (1.3 ± 0.2% vs. 1.2 ± 0.2%) domains of DRG studied.

#### 2.3.6. SOM-Containing UB-PNs in the DRG of Control and GUA-Treated Animals

Another small population of FB^+^ neurons in control DRGs was the one containing SOM (Figure 4m,n; 3.6 ± 0.5%). There were significantly more FB^+^/SOM^+^ neurons in the lumbar DRGs (6.7 ± 0.4%) compared to the sacral/coccygeal ganglia studied (0.5 ± 0.1%). The total number of SOM-containing FB^+^ sensory neurons has not distinctly changed (5.3 ± 0.4%) after bladder instillation of GUA. However, it should be noted that, if compared to the untreated animals, the number of SOM^+^ UB-PNs in sacral/coccygeal DRGs distinctly increased (8.0 ± 1.7%), while the number of such cells visibly decreased (1.0 ± 0.5%) in the lumbar ganglia.

SOM-positive retrogradely traced nerve cells were exclusively of the small type in both experimental groups (Figure 4m,n and Figure 5m,n; short arrows), and they were observed in three domains of DRGs. The majority of SOM^+^ UB-PNs were located in the caudal (53.4 ± 2.2% vs. 51.8 ± 1.9%) as well as in the cranial (44.1 ± 2.2% vs. 45.9 ± 1.9%) regions of the ganglion, while such cells were present in the peripheral ganglionic domain in much smaller number (2.5 ± 0.5% vs. 2.3 ± 0.4%). There were no statistically significant differences in the number of FB^+^/SOM^+^ neurons located in the individual parts of the ganglion between the control group and the GUA-treated animals.

#### 2.3.7. CB-Containing UB-PNs in the DRG of Control and GUA-Treated Animals

CB-containing neurons constituted the smallest population (Figure 4o,p; 2.7 ± 0.4%) of all FB^+^ UB-PNs in the untreated animals, being observed in both the lumbar (3.2 ± 0.3%) as well as the sacral/coccygeal (3.0 ± 0.3%) DRGs. The intravesical administration of GUA caused a statistically significant increase in the number of FB^+^/CB^+^ nerve cells (Figure 5o,p; 8.6 ± 0.5%), especially those located in the sacral/coccygeal DRGs (7.7 ± 0.9%), while the number of such neurons in the lumbar ganglia did not significantly change (2.8 ± 0.4%).

Under physiological conditions, all CB-positive UB-PNs belonged to the subpopulation of medium-sized cells (Figure 4o,p-long arrows). After GUA administration, the majority of FB^+^/CB^+^ neurons were still medium-sized (68,3 ± 1.8%); however, a population of small-sized neurons (Figure 5o,p-short arrows; 32.7 ± 1.8%) has also been observed. In both experimental groups, UB-PNs containing CB were unevenly dispersed in the DRG, mainly in its cranial (48.1 ± 2.7% vs. 47.4 ± 2.2%) and caudal (47.0 ± 2.8% vs. 47.8 ± 2.0%) regions. Additionally, a small population of such cells was observed in the peripheral (3.1 ± 2.7% vs. 3.2 ± 0.2%) as well as the middle (1.8 ± 0.3% vs. 1.6 ± 0.6%) domains of studied ganglia. Details concerning the relative percentages of the individual subpopulations of retrogradely labeled DRG neurons in both the control group and in the group of GUA-treated animals are summarized in Table 2.

## 3. Discussion

The results of the present study clearly indicate that the intravesical application of GUA is followed by profound changes in the chemical coding but not the intraganglionic distribution pattern and the relative frequency of sensory DRG neurons supplying the wall of the porcine urinary bladder. Additionally, the present study confirms that especially the sacro-coccygeal DRGs are an important source of sensory innervation of the urinary bladder in the female domestic pig. 

A thorough discussion regarding the distribution pattern, relative frequency, and chemical coding of DRG neurons supplying the wall of the female porcine urinary bladder was already presented in our previous papers [6,28]. However, it should be stressed that, in both earlier studies, the control groups were slightly different: while in the case of the study focusing on neurochemical characterization of UBPNs [6], no medical procedures were applied to the control animals, in the present study, as well as in an earlier work concerning the influence of botulinum toxin type A (BTX) on the plasticity of SP^+^ bladder-projecting DRG neurons, the control animals were intravesically instilled either with citrate buffer (present study) or with 5% aqueous solution of ethyl alcohol (BTX study) [28]. The aim of the above-mentioned procedures performed in the last two control groups was to ascertain that the differences in the distribution and chemical coding of bladder-projecting DRG neurons, found after the exposure to BTX (previous study) or GUA (this study), were caused by these compounds themselves and not due to factors associated with the mode of their administration. Furthermore, it should be noted that the number, age, body weight, and sex of the animals used as the control groups in mentioned experiments, as well as all the surgical and immunohistochemical procedures applied, were entirely corresponding. Accordingly, no significant differences in the relative number, distribution pattern, chemical coding, and relative frequencies of particular subsets of DRG-UBPNs were observed between the control animals of the present study compared to the control groups used in the previous experiments. Therefore, in the present discussion, we are focusing on the data concerning changes caused by GUA treatment.

The present findings strongly suggest that GUA is a factor evoking distinct adaptational changes in DRG neurons supplying the urinary bladder wall. These changes include modifications of their chemical phenotype and/or alterations in the relative numbers of differently coded populations of bladder-projecting afferent perikarya. The present study has shown that GUA instillation induced a down-regulation in the number of SP-IR and, concomitantly, an up-regulation in numbers of GAL-, nNOS-, or CB-containing urinary bladder afferent neurons, while the numbers of CGRP-, PACAP-, or SOM-containing bladder-projecting sensory cells remained unchanged. Moreover, the expression pattern of these neuropeptides in bladder afferent neurons can be differently up- or down-regulated depending on the spinal cord segmental level at which the parental DRGs were located. In general, it is now accepted that reflex contractions of the bladder are elicited by an activation of parasympathetic preganglionic neurons located in the intermediomedial nucleus of the sacro-coccygeal spinal cord (segments S3 to Cq1 in the pig; see [6]), while an activation of sympathetic preganglionic neurons in the lumbar spinal cord (L3-L6 in the pig; see [6]) has inhibitory effects on bladder smooth muscle activity [29]. Therefore, as there were two distinct “sensory centers” found along the lumbo-sacro-coccygeal DRGs, it is assumable that the functional interpretation of the alterations observed in the present study might be separately derived from the lumbar and sacro-coccygeal DRGs in which GUA-induced changes were observed.

It is well-known that SP released from the bladder afferent nerves at the sacral spinal cord is involved in the mechanoreceptor-mediated micturition reflex. For example, systemic administration of capsaicin in rats, used for the depletion of SP from afferent fibers, resulted in the urine retention or an increased volume/pressure threshold for micturition, implicating an excitatory role of SP in the afferent micturition pathway [30]. Moreover, the central branches of SP-containing, bladder-projecting DRG neurons project to L6-S1 segments of the spinal cord through a bundle (lateral collateral pathway of the Lissauer’s tract) running through lamina I and provide synaptic inputs (either direct or indirect) to the dorsal part of the sacral parasympathetic nucleus [31,32]. It has also been shown that intrathecally applied SP facilitates normal micturition and SP antagonists delivered intrathecally depress this process [33,34]. Another mechanism of SP-induced bladder contraction was observed in guinea pigs, in which SP secreted from afferent sensory terminals activates the release of acetylcholine from the parasympathetic cholinergic nerve fibers in the guinea pig urinary bladder wall [19,35]. Several other reports indicate that SP, present in capsaicin-sensitive bladder afferent neurons, may mediate hyperreflexia [3,36,37], as SP is released from axons of DRG neurons [38,39] upon noxious stimulation in the periphery. All these findings may suggest that this neuropeptide could be involved as an excitatory transmitter in several types of bladder reflexes. On the other hand, SP released from the central endings of afferent neurons located in thoraco-lumbar DRGs has been shown to facilitate the impulse activity of corresponding sympathetic preganglionic neurons of the intermediolateral nucleus [40], which are the source of the sympathetic (inhibitory) innervation of the bladder. It has also been reported that SP released from peripheral afferent nerve endings causes series of local inflammatory responses referred to as neurogenic inflammation [41]. This neurogenic inflammation includes vascular (vasodilation and plasma protein extravasation) [42,43] and nonvascular responses (immune cell activation, leukocyte infiltration, mast cell degranulation, and expression of proinflammatory cytokines, chemokines, adhesion molecules, and cyclooxygenase 2) [44] and has been proposed to play a major role in different diseases of the urinary tract, including cystitis and overactive bladder syndrome [45]. Thus, as may be judged from the above-mentioned studies, it appears possible that the pig SP may also be involved in the regulation of urinary bladder functions at many different levels of this neuraxis. 

In the present study, for the first time, we provided evidence that the number of SP-IR UBPNs slightly decreased in sacro-coccygeal DRGs after intravesical GUA instillation. The opposite results were observed in rats, where long-term administration of GUA increased the expression of SP in DRG neurons [13]. It is probably due to the fact that in peripheral target tissues, sensory and sympathetic nerve terminals compete for nerve growth factor (NGF), a potent stimulator of CGRP and SP expression and release [46,47]. In sympathectomized rats, NGF accumulates in target tissues because there is decreased uptake and sequestration of this protein by sympathetic nerves [48,49]. This elevation of NGF content is apparently sufficient to allow for both an increase in NGF uptake by sensory nerves and overexpression of SP in DRG neurons [13]. One of the putative reasons for the lack of an increase in the number of SP-containing DRG neurons observed in our study was the use a single dose of GUA, while, in the case of previous studies, an increase in SP expression was clearly visible only after repeated administration of GUA over a period of several weeks. Additionally, our results indicate a slight decrease in the relative frequency of this subpopulation of UBPNs. One possible explanation of this phenomenon is that the adrenergic GUA-related block is preceded by a short-term sympathomimetic effect, which is caused by a transient releasing of NE from adrenergic nerve endings [12]. As α1-adrenoceptor activation on terminals of primary sensory neurons releases SP [50], it is possible that the administration of GUA in the first stage causes the release of NE from the sympathetic nerve endings of the bladder wall, which, in consequence, leads to the release of SP from sensory terminals, causing a decrease in the number of SP-positive DRG-UBPNs neurons. As GUA resulted in a decrease in the number of SP-IR bladder sensory neurons, it may be suggested that this neurotoxin, on the one hand, can reduce the excitatory effect of SP at the spinal level, but, on the other hand, can lead to the activation of SP-induced local mechanisms of neurogenic inflammation. However, to confirm these hypotheses, further detailed studies should be conducted.

Results of the present study clearly demonstrated that GUA, instilled into the porcine urinary bladder, was able to evoke a distinct increase in the number of GAL-, nNOS-, and CB-IR sensory neurons supplying this organ. It should, however, be stressed that observed changes varied depending on the spinal cord level at which affected DRG neurons were located. Thus, a strong increase in the number of GAL-, nNOS-, or CB-IR bladder-projecting neurons was predominantly observed in the sacro-coccygeal DRGs. Simultaneously, GUA-induced slight diminishing in the number of GAL-expressing perikarya was present in the lumbar DRGs, while the number of FB^+^ nerve cells containing CB in the same sensory ganglia did not change.

In all animal models of neuropathic pain evoked by peripheral nerve injury [51], not only was GAL upregulated in DRG neurons, but an increase in its release has also been reported in the superficial layers of the spinal dorsal horn [52]. This leads to an activation of the inhibitory GAL1 receptors on putatively glutamate-containing excitatory dorsal horn neurons, resulting in attenuation of pain transmission [53]. On the other hand, a reduction in galaninergic transmission within the dorsal horn appears to increase pain [53], underlining an inhibitory role of high amount of endogenous GAL under pathological conditions. Additionally, it has been reported that GAL, when administrated intrathecally in higher doses, blocked the facilitatory effects of SP and CGRP on the excitability of the nociceptive flexor reflex in rats [54,55]. Based on these results, it may be speculated that the increased expression of GAL in DRG-UBPNs after bladder instillation with GUA contributes to the state of desensitization by antagonizing the effect of SP released from bladder fibers in the spinal cord [56]. GAL has been shown to influence the activity of detrusor muscle in rat bladder as well as to modulate neural transmission at the level of autonomic ganglia [57] and the neuromuscular junctions [58]. Thus, an inhibitory action of GAL on acetylcholine (ACh) release has been suggested in smooth muscle tissues and may also pertain to the urinary bladder [58]. Furthermore, intrathecal administration of GAL in rats delays the onset of micturition, suggesting the inhibitory role of this molecule in the control of this reflex, as reported by Honda and colleagues [59]. Moreover, anti-inflammatory properties of GAL have also been suggested by Callsen-Cencic and Mense [3], who have revealed that GAL is upregulated during bladder cystitis, and it inhibits presynaptically the release of SP and CGRP from capsaicin-sensitive primary afferents in the bladder wall. It has also been reported that GAL may counteract the action of PACAP and nNOS on bladder-projecting afferent cells [60]. In addition to its anti-inflammatory and analgesic properties [61], GAL should also be considered as one of the most important substances involved in neuroprotection, regeneration, and survival of damaged neurons within the central [62] and peripheral [52] nervous system under various pathological conditions. Thus, as may be judged from the above-mentioned data, GAL may have a potent modulatory function within the bladder-controlling neural circuits, which participates in the urinary bladder facilitation and pain transmission. Finally the results of our study may suggest that GUA, by an increase of GAL expression in bladder-projecting sensory neurons, can lead to a diminishing of SP, CGRP, PACAP, or nNOS release from both afferent peripheral terminals in the urinary bladder wall as well as from central nerve terminals within the sacral spinal cord, in this way reducing the excitatory effect of above-mentioned neurotransmitters on the preganglionic neurons forming parasympathetic nucleus as well as their proinflammatory action, which, in turn, may lead to an inhibition of bladder contractions and pain transmission under pathological conditions.

In the present study, we provided evidence that the number of GAL-expressing bladder-projecting sensory neurons distinctly increased in the sacro-coccygeal DRGs after bladder instillation of GUA. However, the mechanism of this increase in GAL expression in bladder DRG neurons under the influence of GUA is unknown. It has been suggested that upregulation of GAL expression in DRG neurons in micturition pathways after spinal cord injury (SCI) may be mediated by changing neurotrophic factors expression such as NGF and brain-derived neurotrophic factor (BDNF) [60]. Since GUA increases the availability of NGF for sensory fibers in the bladder wall, it may lead to an increase in the expression of GAL in the respective sensory neurons. However, this hypothesis requires confirmation in the subsequent studies. Another possible mechanism of GAL overexpression in DRG-UBPNs may be associated with SP-induced neurogenic inflammation. As the inflammation of the urinary bladder induced a marked elevation in the number of bladder afferents expressing GAL [3,63], the possible increase in SP release from afferent terminals of the bladder wall after GUA treatment can lead to overexpression of GAL in DRG-UBPNs by triggering the neurogenic inflammation.

Results of our study demonstrated that bladder instillation with GUA induced a significant increase in the number of nNOS-containing, FB-labeled neurons, especially in the sacro-coccygeal subpopulation of neurons studied. It has previously been shown that NO, being a small, reactive, and gaseous molecule that is able to pass through neuronal membranes easily, may act as a “retrograde transmitter” in the sensory pathways [64]. However, it has been shown that NO may not act as a “classic” transmitter in the nociceptive pathways because antagonists of NOS do not alter baseline nociceptive reflexes. Thus, it has been suggested that NO may play a pivotal role in nociceptive processing in multisynaptic local circuits of the spinal cord [64]. Birder and colleagues [65] demonstrated that the major sites of NO release in the rat bladder are afferent nerves and that NO plays an important role in the facilitation of the micturition reflex evoked by noxious chemical irritation of the bladder as well as is involved, at the spinal level, in the facilitation of the micturition reflex by nociceptive bladder afferents [66,67]. Furthermore, it is well-documented that NO released from the afferent nerve terminals of the urinary bladder in rats participates in the initiation of inflammatory responses and is involved in triggering the painful sensations [65,68]. On the other hand, an inhibitory function of NO in the micturition pathway has also been suggested. Renganathan and colleagues [69] have shown that NO may be an autocrine regulator of sodium currents in C-type DRG neurons, leading, by suppression of both fast and slow sodium trafficking, to DRG hypoexcitability. Additionally, the NO/cGMP pathway may influence the micturition reflex by reducing the excitability of bladder body afferents, thus favoring the bladder filling. In fact, inhibition of NO/cGMP signaling promotes bladder overactivity [70]. This corresponds well with studies showing that NO may have a role in the inhibitory neurotransmission in the porcine trigone and seems to be involved in the endothelium dependent ACh-induced relaxation of pig vesical arteries [71,72]. Thus, it seems probable that, also in the pig, nNOS may alter transmission in neurons constituting circuits regulating urinary bladder functions. As GUA produced a significant increase in the number of nNOS-IR bladder-projecting neurons in the pig, it may be possible that this molecule can increase the excitatory effect of NO at the spinal level, acting on the population of preganglionic parasympathetic neurons, which, in turn, may cause a stimulation of bladder contractions and pain transmission. Additionally, it seems possible that, like GAL, NO may also participate in the adaptive processes enabling the protection and survival of nerve cells under various pathological conditions. However, the physiological relevance, the exact mechanism(s) and place(s) of action(s) of these neuropeptides, and their simultaneous functional interactions in the same neuron in the domestic pig remain to be addressed in detail.

The way in which nNOS-IR is upregulated in bladder afferent neurons following GUA instillation is uncertain but may involve one possible mechanism. It has been shown that an acute [3] or a chronic [66,67] chemical irritation of the rat urinary bladder enhanced the synthesis of NO in primary afferent neurons and its release in the lumbosacral spinal cord, leading to bladder hyperreflexia during inflammation. If GUA instillation into the urinary bladder can lead to SP-induced neurogenic inflammation, it may induce the overexpression of nNOS in DRG-UBPNs, but this hypothesis requires confirmation in subsequent studies.

The main physiological relevance of CB in neurons is to act as a calcium buffer controlling the Ca^2+^ level within the cytoplasm. It is well-documented that CB is responsible for Ca^2+^ homeostasis in neurons and it exerts neuroprotective effects on these cells by preventing them from large fluctuations in free intracellular Ca^2+^ and cell death [73,74,75]. Therefore, it is possible that an increase in the number of CB^+^ DRG-UBPNs probably reflects a defensive reaction of investigated cells, challenged by GUA. Other studies have shown that CB not only functions as a passive Ca^2+^ buffer but also has an active role in neuronal activity [76]. CB was previously found in medium- or small-sized DRG neurons, mostly in the nociceptors of muscles and viscera [77]. Numerous CB-containing small- or medium-sized DRG neurons also contained SP and co-expressed vanilloid receptor type 1 [78]. This strongly suggests that CB may be involved in some aspects of pain transmission, at least in small spinal ganglia neurons. Our study revealed that, in pigs, the number of bladder sensory neurons containing CB distinctly increased in sacro-coccygeal DRGs after bladder instillation of GUA. It probably may lead to changes in CB control action on the Ca^2+^ homeostasis in affected neurons as well as in SP release rate from central afferent branches in the dorsal horn, influencing pain transmission.

Our experiments have shown that the intravesical administration of GUA did not evoke any significant changes in the number of CGRP-, PACAP-, or SOM-IR bladder afferent neurons, either in the lumbar or in sacro-coccygeal DRGs studied.

Previous studies suggested that CGRP, which has no excitatory effect on the vesico-vesical reflex pathway per se [30], is able to facilitate the SP-evoked chemonociceptive reflex. CGRP acts synergistically with SP in the spinal cord [79], and such synergism may result from the CGRP-mediated inhibition of an endopeptidase that degrades SP [80], thus elevating the local concentration of SP at the site of release or by an enhancement of the SP-release from primary afferent fibers in the spinal cord by CGRP, as suggested by Oku et al. [81]. On the other hand, other studies have postulated that exogenous CGRP application or CGRP release from primary afferent nerves relaxes smooth muscle and produces relaxation of the bladder [82,83]. Our experiments have shown that intravesical instillation of GUA did not evoke any significant changes in the number of CGRP-IR bladder afferent neurons either in the lumbar or in the sacro-coccygeal DRGs studied. The mechanism(s) of such resistance against GUA by CGRP-IR DRG-UBPNs remains obscure yet. Previous studies have demonstrated that, after long-term sympathectomy by GUA, the number of trigeminal and DRG neurons expressing CGRP-IR was reduced [84] as a result of GUA-induced elevation of NGF in the target tissue leading to the increase in both the density of CGRP-IR nerve fibers and the releasing of this neuropeptide to the tissue [85].

Although the exact physiological function(s) of PACAP in the lower urinary tract are still unclear, Ishizuka and co-workers [86] reported that PACAP may be involved in the facilitation of spontaneous bladder contractions in rats, due to its excitatory action on the spinal micturition pathway. Moreover, Girard and collaborators [87] suggested that PACAP signaling acts as an agent in the regulation of bladder contractility at the level of the urothelium. Furthermore, it has been shown that PACAP increased bladder smooth muscle tone and potentiated electric field stimulation (EFS)-induced contractions [88], indicating the facilitatory direct effects of PACAP on bladder smooth muscle contractility. It has also been shown that the expression of PACAP in bladder afferent cells in rats increased during chronic cystitis induced by cyclophosphamide treatment [89] or after spinal cord injury [90]. These results indicated that PACAP may represent a principal component of bladder hyperreflexia, leading to an increase in the excitability of sensory neurons participating in the bladder reflex arc. In addition, two supplementary functions have been postulated for PACAP in the micturition reflex pathways, including modulation of nociceptive transmission to the dorsal horn by a direct interaction with N-methyl-D-aspartate (NMDA) receptors [91] and/or modulation of inflammatory responses [92] by downregulation of proinflammatory cytokines synthesis and/or release. It has also been shown in pigs that PACAP produces relaxation in the urinary tract [93]. Two mechanisms have been proposed for PACAP contribution to the smooth muscle relaxation: (1) activation of facilitatory PAC1 receptors located on capsaicin-sensitive afferent terminals, which induces the release of NO, an inhibitory agent that relaxes smooth muscle [94], and (2) activation of presynaptic inhibitory VPAC receptors at autonomic nerve terminals [93,95]. Our findings have shown that GUA did not change the number of PACAP^+^ UB-PNs. In the previous studies, Jongsma Wallin and collaborators [96,97] have demonstrated that enhanced target-derived NGF availability upregulates PACAP expression in small nociceptive DRG cells. Based on these results, it should be assumed that GUA administration into the bladder, because it increases the availability of NGF for the sensory fibers in the bladder, should increase the PACAP expression in the DRG neurons supplying this organ. The lack of such reaction seems to suggest that the PACAP upregulation mechanism in the irritated porcine DRG cells is not directly dependent on the level of available NGF. However, further research is needed to explain the exact mechanisms behind the non-response of UBPNs to GUA administration.

Previous studies have demonstrated that SOM is released into the spinal dorsal horn on peripheral nociceptive stimulation [98] and depresses the firing of dorsal horn neurons activated by noxious stimulation [99]. It has also been shown that SOM exerts a systemic antinociceptive effect [100] and inhibitory action on acute neurogenic and non-neurogenic inflammatory reactions [101]. However, the role of SOM-positive sensory neurons projecting to the urinary bladder remains obscure. GUA bladder instillation did not change the number of SOM^+^ UB-PNs, which may suggest that this neurotoxin does not affect the biological functions of cells using this neuropeptide as their transmitter in the bladder wall.

## 4. Materials and Methods

### 4.1. Laboratory Animals

Investigations were conducted by using twelve immature (8–12 weeks old, 15–20 kg body weight, b.w.) female pigs of the Large White Polish breed. All the animals originated from a commercial fattening farm and were kept under standard laboratory conditions. They were fed standard fodder (Grower Plus, Wipasz, Wadag, Poland) and had free access to water. After the surgical and tracing procedures (see below), the animals were randomly divided into the control (*n* = 6) and GUA-treated group (*n* = 6). As the present study was designated to provide basic data concerning the chemical phenotypes of DRG neurons supplying the urinary bladder wall both under physiological and pathophysiological (GUA-treated animals) conditions, the authors decided to focus on sexually immature female animals in order to exclude any possible influences of reproductive hormones on studied tissues as identified in previous studies [102,103]. The animals were housed and treated in accordance with the rules of the Local Ethics Committee for Animal Experimentation in Olsztyn (affiliated to the National Ethics Commission for Animal Experimentation, Polish Ministry of Science and Higher Education; decision No. 94/2011 from 23 November 2011). All efforts were made to minimize the number of animals used and their suffering.

### 4.2. Anesthesia and Surgical Procedures

All animals were pretreated with atropine (Polfa, Lublin, Poland, 0.04 mg/kg b.w., s.c.) and azaperone (Stresnil, Janssen Pharmaceutica, Beerse, Belgium; 0.5 mg/kg b.w., i.m.) before the beginning of any surgical procedure, and after thirty minutes, sodium pentobarbital (Thiopental, Sandoz, Poland; 0.5 g per animal, administered according to the effect) was given intravenously in a slow, fractionated infusion. The depth of anesthesia was monitored by testing the corneal reflex. 

A mid-line laparotomy was performed in all the animals, and the urinary bladder was gently exposed to administer a total volume of 40 µL of 5% aqueous solution of the fluorescent retrograde tracer FB (Dr K. Illing KG & Co GmbH, Gross Umstadt, Germany) into the right side of the urinary bladder body wall in multiple injections (1 μL of the dye solution per 1 injection with a Hamilton microsyringe equipped with a 26S gauge needle) under the serosa along the whole extension of the urinary bladder dome, keeping a similar distance between the places of injections. To avoid the leakage of the dye, the needle was left in each place of FB injection for about one minute. The wall of the injected organ was then rinsed with physiological saline and gently wiped with gauze. Three weeks later, which is an optimal time for the retrograde tracer to be transported to the DRG and labeled the UB-PNs [6], the control group was treated with intravesical instillation of citrate buffer (pH 4.9; 60 mL per animal). The above-mentioned procedure was performed in the control pigs to ensure that changes in the chemical coding of DRG UB-PNs after the GUA treatment were caused by this biological active substance itself, not due to the factors associated with the administration processes. The GUA-treated group was treated with intravesical instillation of GUA (12 µg of GUA dissolved in 60 mL of citrate buffer per animal, pH 4.9). Ten minutes after the infusion, the contents of the bladder were evacuated, and the catheter was removed. One week after the administration of citrate buffer or GUA, all the pigs were deeply anaesthetized (following the same procedure as describe above) and, after the cessation of breathing, transcardially perfused with freshly prepared 4% paraformaldehyde in 0.1 M phosphate buffer (pH 7.4). Following the perfusion, all the animals were dissected and the bilateral DRG ganglia, together with the spinal cord, were collected from all animals studied (as described previously in detail by Bossowska et al.) [6]. Tissue samples were then postfixed by immersion in the same fixative (10 min at room temperature), washed several times in 0.1 M phosphate buffer (pH 7.4; 4 °C; twice a day for three days), and finally transferred to and stored in 18% buffered sucrose at 4 °C (two weeks) until sectioning.

### 4.3. Sectioning of the Tissue Samples and Estimation of the Total Number of UB-PNs

The right as well as the left DRGs studied were cut with an HM525 Zeiss freezing microtome on transverse 10 µm thick serial sections. Sections were put on chrome alum-gelatin-coated slides, air dried, and examined under the fluorescent Olympus BX61 microscope equipped with a filter set specific for FB. To calculate the relative number of UB-PNs, FB-positive nerve cells were counted in every fourth section (to avoid double-counting of the same neuron; most neurons were approximately 40 μm in diameter) of both the right and the left DRGs of all animals. Only neurons with a clearly visible nucleus were considered. The results were pooled for every experimental animal and statistically analyzed, and the mean number of FB-positive cells was calculated. The total number of FB^+^ nerve cells counted in all DRG from a particular animal as well as the relative frequencies of perikarya in the ganglia belonging to the individual neuronal size classes were presented as mean ± S.D. The diameter of the FB^+^ perikarya was measured by means of an image analysis software (version 3.02, Soft Imaging System, Münster, Germany), and data were used to divide urinary bladder-projecting neurons into the three size-classes: small (average diameter up to 30µm), medium-sized (diameter 31–50 µm), and large afferent cells (diameter > 51 µm), while the statistical analysis was performed with Graph-Pad Prism 8 software (GraphPad Software, La Jolla, CA, USA).

### 4.4. Immunohistochemical Procedure

Single-immunofluorescence was performed on cryostat sections of both the ipsi- and contralateral DRG where the UB-PNs were found, according to a previously described method [6]. Immunohistochemical characteristics of FB^+^ neurons were investigated using primary antibodies against biologically active substances including SP, CGRP, SOM, GAL, PACAP, nNOS, and CB, as the presence of the above-mentioned active substances or their marker enzymes (nNOS) was previously revealed in the porcine UB-PNs [6,28,104]. These primary antisera were visualized by rat- or mouse-specific secondary antisera conjugated to fluorescein isothiocyanate (FITC) or rabbit-specific antibodies conjugated to biotin, and the secondary antibody was then visualized by a streptavidin-CY3 complex. Details concerning all the primary and secondary antibodies used in the present study are listed in Table 3.

Retrogradely labeled and immunostained DRG perikarya were evaluated under an Olympus BX61 microscope (Olympus, Hamburg, Germany) equipped with epifluorescence filter for FB and an appropriate filter set for CY3 or FITC. Relationships between immunohistochemical staining and FB distribution were examined directly by interchanging filters. The images were taken with an Olympus XM10 digital camera (Tokyo, Japan). The microscope was equipped with cellSens Dimension 1.7 Image Processing software (Olympus Soft Imaging Solutions, Münster, Germany).

### 4.5. Estimation of the Chemical Coding of the DRG UB-PNs

To determine the percentages of particular neuronal subpopulations, FB^+^ neuronal profiles were investigated with one combination of the primary antisera and counted in all the ganglia of all animals studied. To avoid double counting of the same neurons, the retrogradely labeled neuronal cells were counted in every fourth section (only neurons with clearly visible nucleus were included). The percentages of FB^+^ neurons immunopositive to a particular biologically active substance or their marker enzyme were pooled in all the animals and presented as mean ± S.D., with n referring to the number of animals. Morphometric data relative to each neuronal class were compared within each animal and among the animals and were analyzed by the Student’s two-tailed t-test for unpaired data using GraphPad PRISM 8.0 software (GraphPad Software, La Jolla, CA, USA). The differences were considered to be significant at *p* < 0.05.

### 4.6. Control of Specificity of the Tracer Staining and Immunohistochemical Procedures

Thorough macroscopic examinations of the sites of FB injections and the tissues adjacent to the urinary bladder were performed before collecting the ganglia. The injection sites were easily identified by the yellow-labeled deposition left by the tracer within the bladder wall. Moreover, the injection sites were also observed in the UV lamp rays in the dark room. The tissues adjacent to the bladder were not found to be contaminated with the tracer. To verify that the tracer had not migrated into the urethra, we analyzed, in cryostat sections and by means of the H&E staining technique (IHC WORLD LLC, Woodstock, NY, USA), possible signs of leakage of the tracer to the junction between the urinary bladder trigone and cranial portion of the urethra. In all the animals studied, no contamination of the tracer was found within the urethra. All these procedures excluded any leakage of the tracer and validated the specificity of the tracing protocol. To test the specificity of primary antibodies and staining reaction, preincubation tests were performed with the sections from DRG ganglia of the control pigs (inactivation of antisera used was performed by incubation of particular antiserum with an excess of appropriate antigen before using to label the control sections; Table 4). 

**Figure 6:**
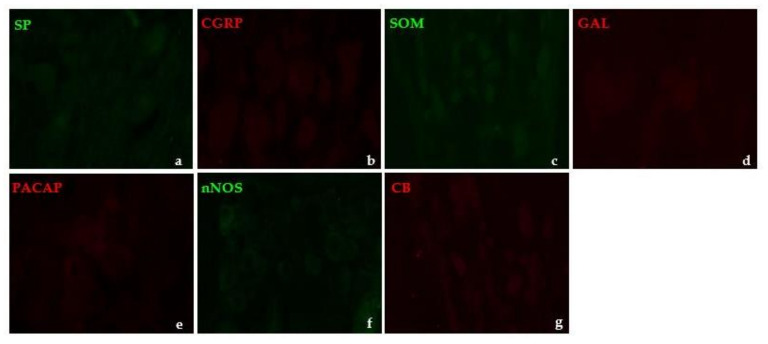
Representative examples of the use of immunofluorescent staining specificity control procedures: (**a**,**b**,**d**,**f**)—omission of primary antibodies, (**c**,**e**,**g**)—replacement of primary antisera by non-immune rat (**c**) or rabbit serum (**e**,**g**).

Overnight preincubation of 1 mL of the primary antiserum at working dilution with 20 µg/mL of the respective peptide (all antigens purchased from Sigma or Abcore, St. Louis, MO, USA or Ramona, CA, USA, respectively) completely eliminated the immunoreaction. The omission and replacement of the respective primary antiserum with the corresponding non-immune serum also served as a negative control. After staining, no immunoreaction was visualized (Figure 6).

## 5. Conclusions

Results of the present study have clearly shown that GUA is able to profoundly change the chemical coding of DRG cells supplying porcine urinary bladder, leading to a decrease in the number of neurons containing SP and a simultaneous increase in the number of GAL-, nNOS- and CB-IR cells. However, GUA was not able to influence CGRP, PACAP, and SOM expression rate in the bladder-projecting sensory cells. Further studies are necessary to elucidate in detail the mode of action and physiological/clinical relevance of this neurotoxin in the bladder-supplying DRG neurons.

## Figures and Tables

**Table 1:** Comparison of neuromere levels at which dorsal root ganglia (DRG) neurons involved in the urinary bladder afferent innervation may be found in various animal species studied so far by means of retrograde tracing.

Species	Sources of Sensory Bladder Innervation
rat	Th13-L2 and L6-S1
guinea pig	L6-S2
pig	L3-L6 and S3-Cq1

**Table 2:** Percentages of FB^+^ neuronal subpopulations in DRG of the control and GUA-treated animals, which simultaneously expressed SP, CGRP, PACAP, GAL, nNOS, SOM, or CB. Data expressed as mean ± SD. Asterisks mark statistically significant differences at * *p* < 0.05, ** *p* = 0.005, *** *p* = 0.0001.

*Experimental* *Groups*	*FB^+^/SP^+^* %	*FB^+^/CGRP^+^* *%*	*FB^+^/PACAP^+^* *%*	*FB^+^/GAL^+^* *%*	*FB^+^/nNOS^+^* *%*	*FB^+^/SOM^+^* *%*	*FB^+^/CB^+^* *%*
*Control pigs*	45.6 ± 1.3	35.6 ± 1.6	26.7 ± 1.6	7.4 ± 0.6	5.4 ± 0.5	3.6 ± 0.5	2.7 ± 0.4
*GUA-treated pigs*	34.6 ± 6.5 *	38.6 ± 0.8	29.9 ± 1.0	12.3 ± 1.0 **	11.9 ± 0.6 ***	5.3 ± 0.4	8.6 ± 0.5 ***

**Table 3:** List of primary antisera and secondary reagents used in this study.

Antigen	Code	Dilution	Host	Supplier
**Primary antibodies**				
SP	8450-0505	1:300	Rat	AbD Serotec; Oxford, UK
CGRP	PC205L	1:9000	Rabbit	Merck Millipore; Temecula, CA, USA
SOM	MAB354	1:30	Rat	Merck Millipore; Temecula, CA, USA
GAL	AB5909	1:4000	Rabbit	Merck Millipore; Temecula, CA, USA
PACAP	T-4465	1:15000	Rabbit	Peninsula; San Carlos, CA, USA;
nNOS	MABN527	1:300	Mouse	Sigma; St. Louis, MO, USA
CB	PC253L	1:6000	Rabbit	Merck Millipore; Temecula, CA, USA
**Secondary reagents**				
Biotinylated anti-rabbit immunoglobulins	E 0432	1:1000	Goat	Dako; Hamburg, Germany
CY3-conjugated streptavidin	711-165-152	1:12000	-	Jackson I.R.; West Grove, PA, USA
FITC-conjugated anti-rat IgG	712-095-150	1:400	Donkey	Jackson I.R.; West Grove, PA, USA
FITC-conjugated anti-mouse IgG	715-096-151	1:600	Donkey	Jackson I.R.; West Grove, PA, USA

**Table 4:** List of antigens used in pre-absorption test.

Antigen	Code	Supplier
SP	S6883	Sigma, St. Louis, MO, USA
CGRP	C0292	Sigma, St. Louis, MO, USA
SOM	S9129	Sigma, St. Louis, MO, USA
GAL	G5773	Sigma, St. Louis, MO, USA
PACAP	A9808	Sigma, St. Louis, MO, USA
nNOS	N3033	Sigma, St. Louis, MO, USA
CB	AC21-2748-P	Abcore, Ramona, CA, USA
